# Abiotic stress responses in plant roots: a proteomics perspective

**DOI:** 10.3389/fpls.2014.00006

**Published:** 2014-01-24

**Authors:** Dipanjana Ghosh, Jian Xu

**Affiliations:** Department of Biological Sciences, NUS Centre for BioImaging Sciences, National University of SingaporeSingapore

**Keywords:** proteomics, abiotic stress, root, adaptive response

## Abstract

Abiotic stress conditions adversely affect plant growth, resulting in significant decline in crop productivity. To mitigate and recover from the damaging effects of such adverse environmental conditions, plants have evolved various adaptive strategies at cellular and metabolic levels. Most of these strategies involve dynamic changes in protein abundance that can be best explored through proteomics. This review summarizes comparative proteomic studies conducted with roots of various plant species subjected to different abiotic stresses especially drought, salinity, flood, and cold. The main purpose of this article is to highlight and classify the protein level changes in abiotic stress response pathways specifically in plant roots. Shared as well as stressor-specific proteome signatures and adaptive mechanism(s) are simultaneously described. Such a comprehensive account will facilitate the design of genetic engineering strategies that enable the development of broad-spectrum abiotic stress-tolerant crops.

## Introduction

Abiotic stresses such as drought, salinity, flood, and cold vastly affect plant growth and metabolism that ultimately disturbs plant life (Bray et al., 2000; Ahmad and Prasad, 2012a,b). This has a negative impact on global crop production since majority of world's arable lands are exposed to these abiotic stress conditions (Rockström and Falkenmark, 2000). Upto 50–70% decline in major crop productivities have been attributed to abiotic stresses on several occasions (Mittler, 2006). For their survival under these stress conditions, plants respond by modifying several aspects in their metabolic cascade (Dos Reis et al., 2012). These response mechanisms help plants to survive during the stress period as well as to recover following cessation of the stress.

Abiotic stress responses in plants occur at various organ levels among which the root specific processes are of particular importance. Under normal growth condition, root absorbs water and nutrients from the soil and supplies them throughout the plant body, thereby playing pivotal roles in maintaining cellular homeostasis. However, this balanced system is altered during the stress period when roots are forced to adopt several structural and functional modifications. Examples of these modifications include molecular, cellular, and phenotypic changes such as alteration of metabolism and membrane characteristics, hardening of cell wall and reduction of root length (Gowda et al., 2011; Atkinson and Urwin, 2012). These changes are often caused by single or combined effects of several abiotic stress responsive pathways that can be best explored at the global level using high-throughput approaches such as proteomics (Petricka et al., 2012).

Proteomics allow global investigation of structural, functional, abundance, and interactions of proteins at a given time point. As a technique proteomics is advantaged over other “omics” tools since proteins are the key players in majority of cellular events. In addition to its capability of complementing transcriptome level changes, proteomics can also detect translational and post-translational regulations, thereby providing new insights into complex biological phenomena such as abiotic stress responses in plant roots (Gygi et al., 1999b; Salekdeh et al., 2002). In this review, proteomics studies on root responses against drought, salinity, flood, and cold are discussed with an aim to highlight shared as well as stressor specific protein classes altered due to stress conditions.

## Tools and techniques for plant proteomic analyses

Advances in high-throughput proteomics helped to address complex biological questions in various species. However, plant proteomics still have to deal with several technical challenges. For instance good sample quality is one of the critical factors for successful proteomic experiments and is challenging to obtain from plant tissues. An enriched level of proteases and oxidative enzymes in plant tissues make it extremely difficult to extract stable protein mixtures. Moreover, secondary metabolites produced in plant cells often interfere with subsequent protein fractionation and downstream analyses. Hence it is notoriously difficult to extract complete and representative protein population from plant tissues. Additional hindrance comes from the cell wall that is difficult to fragment. Use of TCA (Trichloroacetic acid)—acetone precipitation and phenol extraction method helped to overcome the above challenges to certain extent (Isaacson et al., 2006). However, optimizations to certain experimental conditions are still essential considering the heterogeneity between species. In addition, low protein content in plant cells has been another major limitation to effective extraction of proteins from plants.

Protein extraction is usually followed by protein separation and identification that can be achieved with the use of two-dimensional electrophoresis (2-DE) (Wittmann-Liebold et al., 2006) or liquid chromatography coupled with tandem mass spectrometry (LC-MS/MS) (Fournier et al., 2007). Although the merits of gel based separation techniques have been debated (Gygi et al., 2000) when compared to the LC-based shotgun approach, both separation strategies are being widely used with their own advantages and disadvantages. Gel based approaches are widely used for their simplicity, reproducibility, wide molecular weight coverage, and detection of post-translational modifications. However, careful manual editing is essential to obtain high precision especially for comparative proteomics. Additionally narrow pI range coverage and inability to detect low abundant proteins limited the use of this technique for broad protein mapping (Gygi et al., 2000). Protein spots obtained upon separation on a 2D gel are then trypsin digested into peptides for further protein identifications. On the contrary, LC-based separation strategy uses digestion before separation in most of the cases. This separation system covers broad molecular weight range along with identification of low abundant proteins (Fournier et al., 2007).

**Figure 1:**
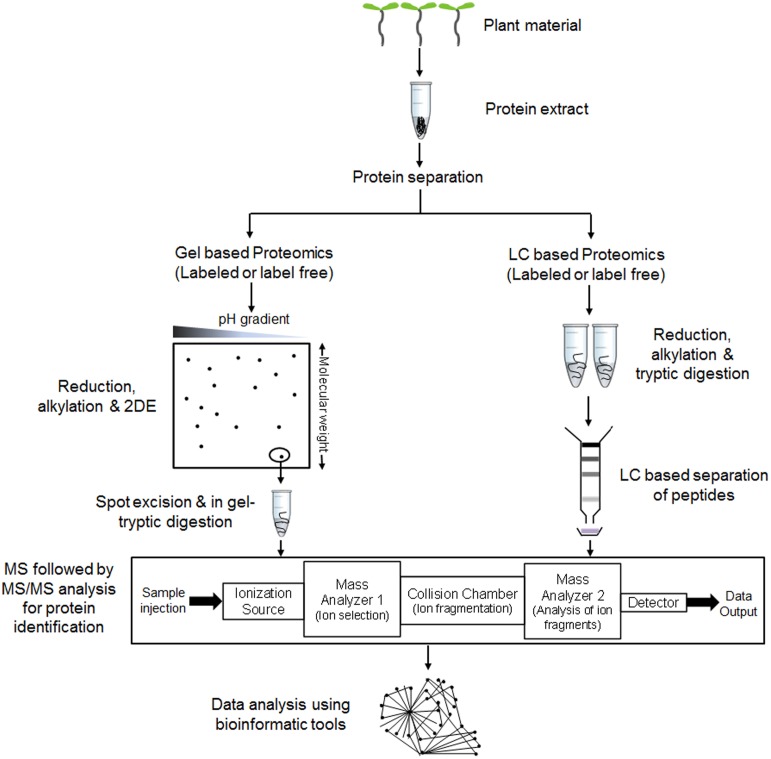
**A general workflow of comparative proteomic experiments in plants**. Proteins are extracted and subjected to separation via gel (e.g., 2DE or 2 Dimensional gel electrophoresis) or non-gel (e.g., LC or liquid chromatography) based approaches. Reduction, alkylation and digestion are performed before or after the separation step as per the requirement to convert protein mixtures into peptides. Separated peptides are analyzed through mass spectrometer (MS) followed by tandem MS (MS/MS) for determining protein identity. The detected protein list is then used for data analysis using various bioinformatic tools.

Protein identification followed by separation has mainly outstretched with the advances in mass spectrometry (MS). Firstly, breakthroughs in soft ionization methods such as matrix assisted laser desorption ionization (MALDI) (Tanaka et al., 1988) or electrospray ionization (ESI) (Yamashita and Fenn, 1984) and secondly peptide fragmentation by collision-induced dissociation (CID) in tandem MS (Stephenson and McLuckey, 1998) helped to achieve excellent coverage. Peptides identified through MS and MS/MS is finally searched against particular protein database to obtain a list of proteins. Recent advances in identification of qualitative changes like post-translational modifications allow differentiating between identical peptide mass and its modified variants which are important from biological perspective. Along with the qualitative changes, spatiotemporal variations in quantitative biomolecule ratios within a cell are also of high significance for better explanation of molecular events. Introduction of LC-MS based tagging approaches such as isotope-coded affinity tags (ICAT) (Gygi et al., 1999a), stable isotope labeling by amino acids in cell culture (SILAC) (Martinović et al., 2002; Ong et al., 2002, 2003; Ibarrola et al., 2004), isobaric tags for relative and absolute quantitation (iTRAQ) (Ross et al., 2004; Choe et al., 2007; Ghosh et al., 2011, 2013) helped to explore this field by relatively quantifying proteins or peptides at a global level. Emergence of statistically robust label free quantitative approach is also helping quantitative proteomics research to analyze large number of clinical samples (Chelius et al., 2003; Liu et al., 2004; Wu et al., 2006). Hence, with the existing as well as ongoing advances in the MS field, proteomics are expected to provide improved ways to reveal biological information. Figure 1 describes a typical workflow of proteomic studies on plant tissues.

Since genome sequences of many plant species especially crops are still lacking, protein databases available for the model plants such as Arabidopsis and rice are currently used as reference databases for analyzing data obtained from other plant species. Therefore, up to now proteomics data analysis in plants is not well-optimized. However, further improvements are expected with increasing number of genome sequences made available for various plant species.

## Proteomics overview on abiotic stress responses in plant roots

Abiotic stress biology research in plants has been enriched with a broad range of transcriptomic and proteomic studies that provide comprehensive information on alteration of gene expression and proteome profile during and following stress conditions (Hakeem et al., 2012; Mizoi et al., 2012). At the transcript level, abiotic stress responses were mainly studied from 30 min to 1 day after stress induction (Kilian et al., 2007). Whereas investigations carried out with comparative proteomic approaches were often performed on plants exposed to a particular stressor for at least 1 day. The time lapse between transcriptomic and proteomic studies was probably determined based on the time required for translation process in eukaryotes (Berthelot et al., 1973). Approximately 50% of the genes responsive to flood, salinity and extreme temperatures were found to encode transcriptional regulators (Kilian et al., 2007; Mizoi et al., 2012). Thus, transcription factors were immensely highlighted as regulators of abiotic stress responses at RNA level studies (Jaglo-Ottosen et al., 1998; Kasuga et al., 1999; Seki et al., 2001; Kilian et al., 2007; Mizoi et al., 2012). However, identification of low abundant proteins such as transcription factors were limited by the use of gel based separation techniques in proteomic studies. Nevertheless, proteomic studies have led to the identification of various abiotic stress responsive proteins, some of which might be downstream effectors of the transcription factors identified at the transcriptional level. Moreover, MS-based proteomics allow isoform specific protein identification and hence are able to differentiate specific and shared functions within a protein family. This level of detection is often not possible in transcriptomic studies. Thus, proteome-wide identification and functional analysis of proteins provide additional insights into the findings obtained at the transcriptional level and thereby allow a better understanding of abiotic stress response pathways in plants. Table 1 summarizes various proteomic studies performed with roots of different plant species grown under drought, high salinity, flooding or cold condition. The proteome wide alterations during root responses to each stressor are discussed in detail in the following sub-sections.

**Table 1:** **A summary of comparative proteomic analyses performed with roots treated with different abiotic stresses. Plant species, stress treatment conditions, proteomic approaches, and protein classes identified in these studies are described**.

**Species**	**Treatment**		**Proteomics approach**	**References**	**Protein classes identified**
	**Condition**	**Duration**			
**DROUGHT**					
Soybean	10% PEG 6000	4 days	2-DE	Mohammadi et al., 2012a	Metabolic enzymes Lignin biosynthesis related enzymes Small G-protein family members Osmolytes and trans-membrane H_2_O-channels ROS scavengers Molecular chaperones Proteosomal factors Protease inhibitors Proteolytic enzymes Translation factors
	Stop watering	5 days	2-DE	Alam et al., 2010	
	PEG 6000	4 days	2-DE	Toorchi et al., 2009	
Wild watermelon	Stop watering	–	2-DE	Yoshimura et al., 2008		
	Stop watering	–	2-DE	Yoshimura et al., 2000		
Rapeseed	Stop watering	1–7 days	2-DE	Mohammadi etal., 2012b	
Wheat	18% PEG 6000	–	2-DE	Demirevska etal., 2008	
Sugarcane	Stop watering	3 weeks	1-DE, 2-DE	Jangpromma et al., 2010		
**SALINITY**					
Rice	150 mM NaCl	–	2-DE	Cheng et al., 2009	Plasma membrane receptors Ca^++^ signaling protein Kinases Ethylene receptors ROS scavengers Ion channel proteins Membrane proteins Metabolic enzymes Enzymes involved in ETC and ATP synthesis
	200 mM NaCl	1, 3, and 6 h	2-DE	Zhang et al., 2009		
	150 mM NaCl	10 and 24 h	2-DE	Chitteti and Peng, 2007		
	5 μ M ABA	48 h	2-DE	Li et al., 2010		
	100 mM NaCl	2 weeks	2-DE	Malakshah et al., 2007		
Wheat	200 mM NaCl	24 h	2-DE	Peng et al., 2009		
	201 mM NaCl	24h	2-DE	Wang et al., 2008	
Arabidopsis	150mM NaCl	6 and 48h	2-DE	Jiang et al., 2007	
Maize	25 mM NaCl	1 h	2-DE	Zörb et al., 2010	
	100mM NaCl	9 days	2-DE	Zörb et al., 2004	
Wild tomato	200 mM NaCl	–	2-DE	Zhou et al., 2011	
Pea	150 mM NaCl	6 weeks	2-DE	Kav et al., 2004	
Creeping bentgrass	Nacl	28 days	2D DIGE	Xu et al., 2010	
Sugar beet	900 mM Nacl	–	2D DIGE	Yang et al., 2012	
Cucumber	50 mM Nacl	3 days	2-DE	Du et al., 2010	
Barley	250 mM Nacl	13 days	2-DE	Witzel et al., 2009	
	200 mM NaCl	5 days	2-DE (gradient)	Sugimoto and Takeda, 2009	
**FLOOD**					
Wheat	Submerged in water	2 days	2-DE	Kong et al., 2010	Disease/defense-related proteins Metabolic enzymes Molecular chperones Cytoskeleton proteins Cell wall biosynthesis related proteins Signaling molecules Proteins related to *de novo* protein synthesis
Soybean	Submerged in water	12–48 h	2D DIGE	Nanjo et al., 2010		
	Submerged in water	1–3 days	2-DE	Salavati et al., 2012		
	Submerged in water	12 h	2-DE	Komatsu et al., 2013		
**COLD**					
Rice	10°C	24 and 72 h	2-DE	Lee et al., 2009	Primary metabolism associated enzymes Antioxidants Molecular chaperones Proteins involved in cellulose biosynthesis Membrane proteins Signal transduction molecules Defense-related proteins
	5°C	48 h	2-DE	Hashimoto and Komatsu, 2007		
	15°C, 10°C, and 5°C	24 h	2-DE	Hashimoto et al., 2009		
Chicory	<5°C	–	2-DE	Degand et al., 2009		
Maize	10°C	7 days	2-DE	Kollipara et al., 2002		
Poplar	4°C	4, 7, and 14 days	2-DE	Renaut et al., 2004		
Pea	6–8°C	11 days	2-DE	Dumont et al., 2011		

### Drought stress

Prolonged water deficit in the soil causes drought, one of the prevalent abiotic stresses that vastly affect the metabolic and physiological functions of a growing plant. Being the primary organ that detects changes in soil condition, root plays an important role in the drought response. Over the last decades, mechanisms involved in the drought response in roots have been extensively studies at the protein level using comparative proteomic approaches. These proteomic studies have linked various functional protein classes to drought responses.

Carbon/nitrogen metabolism related proteins such as triosphosphate isomerase, malate dehydrogenase, α-mannosidase, UDP-sugar pyrophosphorylase, NADP-malic enzyme, phosphoglucomutase, and UDP-glucose-6-phosphate dehydrogenase, were reported to be more abundant in roots of soybean (Toorchi et al., 2009; Alam et al., 2010; Mohammadi et al., 2012a), wild watermelon (Yoshimura et al., 2008), and rapeseed (Mohammadi et al., 2012b) 1 day after drought treatment. This reflected an increased energy demand as well as enhanced cellular activities in the root tissues at this stage of the stress. Simultaneously, a relative increase in root growth rate was observed, which was further supported by the abundance of root growth related small G-protein family members such as Ran GTPases (Yoshimura et al., 2008). Such root elongation could be the indication of an effort by the root to absorb water from deep soil layers. To replenish water deficit within the systems, roots also developed other mechanisms such as enhanced pumping of protons into vacuoles (Mohammadi et al., 2012b). As a signature of this, osmolytes and trans-membrane water-channel proteins such as vacuolar-type H^+^-ATPases and plasma-membrane associated cation-binding protein 1 were found to be synthesized and stored at high levels in drought-induced plant species (Ishitani et al., 1995).

During drought stress, photosynthetic electron transport chain was markedly suppressed and as a consequence the excess excitation energy was driven towards the production of reactive oxygen species or ROS. To counteract the harmful effects of these ROS, several ROS scavengers were induced during drought stress. These include dehydrins, dehydroascorbate reductase, quinine reductase, γ-glutamyl cysteine synthetase, and glutathione S-transferases as observed from proteomic studies pursued on soybean (Toorchi et al., 2009; Alam et al., 2010; Mohammadi et al., 2012a), wild watermelon (Yoshimura et al., 2008), tomatoes (Shalata et al., 2001; Mittova et al., 2004), sunflower (Di Baccio et al., 2004), and other plant species (Mishra and Das, 2003). Additionally, increased levels of molecular chaperones, such as heat shock proteins, were detected in roots of sugar beet (Hajheidari et al., 2005), wheat (Demirevska et al., 2008), wild watermelon (Yoshimura et al., 2000), and sugarcane (Jangpromma et al., 2010) under drought treatment. These proteins may play a cytoprotective role in roots by preventing aggregation and assisting refolding of non-active proteins (Hartl, 1996).

Evidence from several proteomic studies showed that roots respond to drought stress using mechanisms similar to those occurring in damaging cells. For instance, enhanced levels of proteosomal factors were detected in drought-tolerant varieties of rapeseed seedlings (Mohammadi et al., 2012b). Similarly, in watermelon roots several proteolytic enzymes known to facilitate degradation of irreversibly damaged proteins were found to be induced by drought (Yoshimura et al., 2008). These include leucine aminopeptidases, ubiquitin family proteases, cysteine proteases, and multicatalytic endopeptidase (Yoshimura et al., 2008). Moreover, defense-related proteins and protease inhibitors, such as phloem serpin-1, Knotted 1 (kn1), pentatrico peptide repeat (PPR) protein (Yoo et al., 2000; Alam et al., 2010), NB-LPR or Nucleotide binding domain, leucine-rich repeat protein (Yu et al., 1998), and jasmonate-inducible proteins (Mohammadi et al., 2012b), were also found in abundance in roots of *Cucurbita maxima*, soybean, wild, and cultivated tomatoes, respectively. Together, these findings strongly suggest that defense-related proteins and proteins reported to regulate programmed cell death (PCD) are also involved in the response of roots to drought stress.

As soon as drought conditions are withdrawn, plants enter the recovery phase that can be characterized by certain proteome signature. For example, enhanced levels of actin isoform B was observed in the leaf, hypocotyl, and root of drought-affected soybean seedlings (Mohammadi et al., 2012a), indicating that actin is involved in repairing injured membranes following drought stress. Moreover, structural components of the cell wall were also altered in the root during the drought recovery phase. Examples of these include proteins related to lignin biosynthesis, such as caffeoyl-CoA 3-O-methyl-transferases and class III plant peroxidases that were found to be induced by drought in wild watermelon (Yoshimura et al., 2008) and maize (Degenhardt and Gimmler, 2000) roots and thereby suggesting enhanced lignin production. Increased amount of lignin builds the mechanical strength of cell wall and thereby protects roots against the dry soil (Yoshimura et al., 2008). In addition, cell wall modification is also used to minimize water loss and cell dehydration, thus helping plants to resist and recover from drought upon availability of water.

### Salt stress

Salt stress is developed from excessive concentrations of salt, especially sodium chloride (NaCl) in soil. Root is the primary organ of exposure and hence responds rapidly. Several proteomic-based investigations have provided new insight into root responses and adaptation against high salinity.

Proteins involved in signal perception were found to be higher in abundance at the early stage of salt stress (Zhao et al., 2013). These include: (1) receptors in the plasma membrane (PM) or in the cytoplasm, (2) G protein, (3) Ca^++^ signaling protein or Ca^++^ binding protein, (4) phosphoproteins involving activation of kinase cascade, and (5) ethylene receptors. Receptor protein kinases (RPKs) in rice roots (Cheng et al., 2009; Zhang et al., 2009), transforming growth factor β receptor-interacting protein in wheat roots (Peng et al., 2009) and small GTP binding proteins in wheat (Wang et al., 2008), Arabidopsis (Jiang et al., 2007), and rice (Chitteti and Peng, 2007; Zhang et al., 2009) roots were found to be rapidly induced by high salinity. Ca^++^ signaling related proteins such as calmodulin (CaM) and calreticulin (CRT) (Jiang et al., 2007; Cheng et al., 2009; Li et al., 2010; Zörb et al., 2010) were also found in higher levels during salt stress. In addition, salt responsive protein kinase cascade was activated in roots of rice (Chitteti and Peng, 2007), wheat (Peng et al., 2009), maize (Zörb et al., 2004), wild tomato (Zhou et al., 2011), pea (Kav et al., 2004), and creeping bentgrass (Xu et al., 2010). Moreover, 14-3-3 family proteins, such as GF14a and GF14b in rice (Malakshah et al., 2007), 14-3-3 proteins in sugar beet (Yang et al., 2012), and 14-3-3 like protein A in wheat (Wang et al., 2008) were more abundant in roots exposed to high salinity. 14-3-3 proteins are positive regulators of H^+^-ATPase activity, which is known to initiate the stress responses (Malakshah et al., 2007) by modulating the electro-chemical gradient across the PM (Finnie et al., 1999).

As adaptive responses to salt stress, root triggers several cellular and molecular events such as (1) alteration in carbohydrate and energy metabolism, (2) changes in ion homeostasis and membrane trafficking, (3) ROS scavenging, and (4) dynamic reorganization of cytoskeleton and redistribution of cell wall components.

Alteration in carbohydrates and energy metabolism under salinity can be addressed by the high abundance of enzymes involved in glycolysis, TCA cycle, electron transport chain (ETC), and ATP synthesis (Kav et al., 2004; Zörb et al., 2004; Chitteti and Peng, 2007; Peng et al., 2009; Du et al., 2010; Manaa et al., 2011). For example, NADH (reduced form of nicotinamide adenine dinucleotide) dehydrogenases, CCOs (cytochrome c oxidases), and ATP synthase subunits were found to be induced by salt stress in the root of many plant species (Chitteti and Peng, 2007; Jiang et al., 2007; Wang et al., 2008; Peng et al., 2009; Yang et al., 2012). Moreover, glycolytic enzymes such as fructose bisphosphate aldolase (FBPA), triosephosphate isomerase, glyceraldehyde 3-phosphate dehydrogenase (GAPDH), phosphoglycerate kinase (PGK), phosphoglycerate mutase, enolase, pyruvate decarboxylase, and alcohol dehydrogenase (ADH) were found to be more abundant in roots of rice (Chitteti and Peng, 2007; Cheng et al., 2009; Li et al., 2010), wheat (Wang et al., 2008; Peng et al., 2009), maize (Zörb et al., 2004, 2010), tomato (Manaa et al., 2011), pea (Kav et al., 2004), and cucumber (Du et al., 2010) under salt treatment. A similar trend was observed for TCA cycle enzymes, including pyruvate dehydrogenase, dihydrolipoamide dehydrogenase, aconitate hydratase, isocitrate dehydrogenase, succinyl-CoA ligase, and malate dehydrogenase as reported in proteomic studies with Arabidopsis (Jiang et al., 2007), rice (Chitteti and Peng, 2007; Li et al., 2010; Nam et al., 2012), wheat (Wang et al., 2008; Peng et al., 2009), wild tomato (Zhou et al., 2011), pea (Kav et al., 2004), and cucumber (Du et al., 2010). Enhanced levels of these primary metabolism related enzymes thus indicates that adequate energy is a prerequisite for roots to deal with high salinity.

Salt stress is known to increase Na^+^/K^+^ ratio in the root that leads to cell dehydration and ion imbalance (Tester and Davenport, 2003; Cavalcanti et al., 2007; Munns and Tester, 2008). To block or reduce cell dehydration and maintain ion homeostasis, plant roots have developed several strategies to enhance Na^+^ exclusion and decreases Na^+^ entry (Tester and Davenport, 2003). These strategies were implemented by modulating the activity of ion channels, V-ATPases and several salt responsive transporters. Among the ion channel proteins, enhanced levels of voltage-gated potassium channel (VGPC) in wheat roots (Peng et al., 2009), voltage-dependent anion channel protein (VDAC) in roots of maize and wild tomatoes (Zörb et al., 2010; Zhou et al., 2011), and lowered level of cyclic nucleotide-gated channel (CNGC) in wheat roots (Wang et al., 2008) were detected, in agreement with their roles in balancing Na^+^/K^+^ ratio. Most subunits of V-ATPases were induced in the root proteomic studies performed under salinity stress. These include two isoforms of V-ATPase subunit A in maize (Zörb et al., 2004) and cucumber (Du et al., 2010), five isoforms of subunit Ein rice (Cheng et al., 2009), wheat (Wang et al., 2008), and pea (Kav et al., 2004), as well as a subunit in sugar beet (Yang et al., 2012). ABC transporters in wheat roots were found to be more abundant during salt stress (Wang et al., 2008; Peng et al., 2009), thus demonstrating their significant roles in salinity response. Alongside, several membrane associated proteins, such as annexin, remorins, PM polypeptides, and membrane steroid binding proteins, were also identified to be responsible for balancing the ion gradient throughout the membrane during salt stress (Lee et al., 2004; Peng et al., 2009).

As a result of water deficit in roots during salt stress, excess amount of reactive oxygen species (ROS) was produced (Miller et al., 2010). Consequently, various ROS scavengers were found to appear at high levels to decrease the excess ROS levels. Proteomic studies on several salt responsive species such as Arabidopsis (Jiang et al., 2007), wheat (Wang et al., 2008; Peng et al., 2009), wild tomato (Zhou et al., 2011), pea (Kav et al., 2004), cucumber (Du et al., 2010), salt cress (Zhou et al., 2010), and creeping bentgrass (Xu et al., 2010) showed abundance of superoxide dismutase (SOD), indicating its role as a key ROS scavenger during this stress condition. Catalase pathway was found to be deactivated in roots of barley (Witzel et al., 2009) and cucumber (Du et al., 2010) after 7 days of salt stress. By contrast, peroxiredoxin and thioredoxin levels were found to be induced by salt stress in roots of maize (Zörb et al., 2010), cucumber (Du et al., 2010), salt cress (Zhou et al., 2010), rice (Zhang et al., 2009; Nam et al., 2012), and wild tomato (Zhou et al., 2011) respectively. These findings together indicate that the above-mentioned enzymes play key roles in protecting root cells from salt-induced oxidative damage. Moreover, proteomic studies with different tissues/organelles from different species revealed dynamic changes in isoforms of various ascorbate-glutathione (AsA-GSH) cycle-related enzymes. For example, in roots of Arabidopsis (Jiang et al., 2007), rice (Chitteti and Peng, 2007; Nam et al., 2012), wheat (Wang et al., 2008; Peng et al., 2009), barley (Sugimoto and Takeda, 2009), and salt cress (Zhou et al., 2010), most glutathione S-transferase (GST) isoforms were found to be salt-inducible, with the exception of GST11 (Jiang et al., 2007), suggesting that the glutathione peroxidase (GPX/GST) pathway was activated to combat salinity. Furthermore, in Arabidopsis root, salt responsive peroxidase (POD) isoforms showed an initial decrease in abundance under salinity stress followed by an increase (Jiang et al., 2007).

While recovering from salt stress, cytoskeleton organization and cell wall components are commonly altered to maintain cell turgor by adjusting cell size (Ndimba et al., 2005; Li et al., 2011). Basic cytoskeleton components such as actin (Chitteti and Peng, 2007; Jiang et al., 2007; Xu et al., 2010), tubulin (Ndimba et al., 2005; Jiang et al., 2007; Katz et al., 2007; Peng et al., 2009; Pang et al., 2010), and other cytoskeleton-related proteins such as some actin-binding proteins (ABPs) (Yan et al., 2005), kinesin motor (Chitteti and Peng, 2007; Sobhanian et al., 2010), myosin (Cheng et al., 2009; Peng et al., 2009), and xyloglucan endotrans-glycosylase (XET) hydrolases (Zörb et al., 2010) were found to have altered abundance during recovery from salinity stress. In addition, changes in cytoskeleton organization and cell wall components were reported to be associated with other physiological responses occurred during salinity stress. One of the examples of these physiological changes include the control of cell expansion and morphology by co-migration of tubulin and P-type ATPases (Campetelli et al., 2005) to connect with the PM (Dryková et al., 2003). Another example includes XETs that enabled wall loosening required for cell expansion by nicking and re-ligating the inter-microfibrillar xyloglucan chains (Fry et al., 1992).

### Flooding stress

Heavy or continuous rainfall in areas with poorly drained soil causes flood, one of the most severe environmental stresses affecting plants, in particular at their early growth stages. Soybean, wheat, barley, and maize are categorized as flood-sensitive whereas rice is an example of flood-tolerant species (Komatsu et al., 2012). The hypoxic environment formed due to submerged state during flooding stress, affects aerobic respiration (Bailey-Serres and Voesenek, 2008), which leads to a boosted production of ATP and regeneration of NAD^+^ through anaerobic respiration (Gibbs and Greenway, 2003). Under this oxygen deprived condition, protein synthesis is hampered as well, since translation is a tremendously energy-intensive process (Nanjo et al., 2010). In order to cope with this stress, plants need to adopt several changes in their gene expression profiles as well as at cellular protein levels.

Proteomic studies with plants grown under flooding stress conditions identified many differentially regulated proteins, which provide insights into flood-induced response mechanisms. One of the early root responses against flood stress could be attributed to the altered abundance of proteins involved in primary metabolism, energy production and secondary metabolism. For example, several proteins involved in the primary metabolism of sugars and polysaccharides (UDP-glucose dehydrogenase, UGP, β-glucosidase G4 and rhamnose synthase), amino acids (aspartate aminotransferase), and lipids (lipoxygenase) were induced as early flood responsive proteins (Jackson and Ram, 2003; Nanjo et al., 2010). On the contrary, phenolics synthesis pathway enzymes such as isoflavonoid synthesis enzyme dihydroflavonol reductase, phenylalanine ammonia-lyase, 6'-deoxychalcone synthase were found to be decreased in abundance. Secondary metabolism related proteins such as S-adenosylmethionine synthetase, caffeic acid 3-O-methyltransferase, and dihydroflavonolreductases were also declined in level under flood stress as observed in a study with flooded soybean seedlings (Nanjo et al., 2010). These enzymes are members of the phenlypropanoid pathway. A decreased level of these enzymes thus justifies the reason behind inhibited pigmentation during flooding as a way of energy conservation.

Another change induced by flooding was the decrease of cell wall synthesis. For example, rhamnose synthase, a key component of plant cell wall was decreased in abundance as observed in a study with flooded soybean seedling (Nanjo et al., 2010). A proteomic study on cell wall specific proteins in wheat roots also revealed lower levels of methionine synthase, β-1, 3-glucanases, and β-glucosidase suggesting that roots of wheat seedlings respond to flood stress by coordinating methionine assimilation and cell wall hydrolysis, thus restricting cell growth. Together these data suggest that, during flood condition, cell wall synthesis is inhibited to reduce energy consumption. On the other hand, restriction of cell wall polysaccharide hydrolysis helps to preserve carbohydrate resources in the cell wall, which can support plant survival under flooding conditions (Kong et al., 2010).

ROS scavengers such as peroxidase, ascorbic peroxidase, and superoxide dismutase were found to be lower in abundance during flood (Shi et al., 2008; Komatsu et al., 2010, 2012), since generation of ROS is limited during the hypoxic condition. Proteins involved in proteolysis, protein folding, and storage was found to be high in abundance that indicates their probable involvement in excluding damage induced non-active proteins. One of the examples of these categories include heat shock proteins that function as a molecular chaperone in variety of cellular processes such as prevention of protein aggregation, translocation of nascent chains across membranes, assembly, or disassembly of multimeric protein complexes, and targeting proteins for lysosomal or proteasomal degradation (Komatsu et al., 2010, 2012). Moreover, disease/defense-related proteins such as glycosylated polypeptides, α-amylase/subtilisin inhibitors and chitinases were enhanced in abundance in wheat roots during flood condition (Kong et al., 2010). Among these, α-amylase/subtilisin inhibitors function in defense against micro-organisms (Yamasaki et al., 2006) and chitinase is involved in a defense mechanism against pathogens as well as abiotic stresses (Shibuya and Minami, 2001). Higher abundance of these proteins in the root thus indicated that molecular processes such as protein folding and degradation are involved in plant adaptive responses toward unfavorable environmental condition. This kind of adaptation gradually leads to damage and exclusion of root tip cells that were injured due to the flooding stress (Zhang and Glaser, 2002; Yanagawa and Komatsu, 2012).

Simultaneously, roots develop ways for flood recovery that can be characterized by certain proteome-wide changes (Dubey et al., 2003; Komatsu et al., 2012; Salavati et al., 2012). Cytoskeleton associated proteins such as actin isoform B, were enhanced in abundance as observed in post-flooded roots, suggesting cell wall expansion for root elongation (Wasteneys and Galway, 2003). Alongside, cell wall biosynthesis takes place during the recovery period and needs the production of deoxy-sugars through the dTDP-glucose 4-6 dehydratase reaction (Seifert, 2004; Chen et al., 2011). Proteins related to secondary metabolism such as S-adenosylmethionine synthase were found to be high abundant in the post flooding recovery period although they were lowered in abundance during flooding. This indicates its requirement for metabolites formation in order to overcome the flooding effect (Hesse et al., 2004). Protein levels for signaling molecules such as phosphatidyl ethanolamine binding proteins or PEBPs was enhanced during the flood recovery as well. This class of proteins being the regulator of various signaling pathways, control growth and differentiation (Salavati et al., 2012). Abundance levels for this group of proteins were found to be gradually decreasing with progression of recovery time which leads to the indication that these proteins were used up during the recovery process (Karlgren et al., 2011; Salavati et al., 2012). Moreover during post-flooding recovery, *de novo* protein synthesis was activated with an increased demand for synthesis of immunophilins, possessing peptidyl-prolylcis-trans isomerase activity (Romano et al., 2005; Salavati et al., 2012). Together, these findings suggested that re-organization of cytoskeleton, alteration of cell wall structure, synthesis of S-adenosylmethionine associated secondary metabolites and *de novo* protein synthesis are the key cellular processes responsible for the recovery of root from flooding stress (Salavati et al., 2012; Komatsu et al., 2013).

### Cold stress

Cold or low-temperature stress is one of the major abiotic stresses that severely affect plant growth and survival. Chilling (<20°C) or freezing (<0°C) temperatures can induce ice formation in plant tissues, leading to cellular dehydration (Chinnusamy et al., 2007). To cope with this adverse condition, plants adopt several strategies such as producing more energy by activation of primary metabolisms, raising the level of antioxidants and chaperones, and maintaining osmotic balance by altering membrane structure (Uemura et al., 1995; Prasad, 1996; Sharma et al., 2005).

Comparative proteomics performed on cold tolerant and sensitive plants helped to understand the overall response as well as recovery mechanism against cold stress. For instance, activation of metabolic processes was observed in rice roots upon 24–72 h of chilling stress as indicated by the enhanced levels of several metabolism-associated proteins (Lee et al., 2009). These include a group of carbohydrate metabolism enzymes, such as phosphogluconate dehydrogenase, NADP-specific isocitrate dehydrogenase, fructokinase, and cytoplasmic malate dehydrogenase. In addition, higher abundance of pyruvate orthophosphate dikinase precursors (PPDK), aconitate hydratase, glycine dehydrogenase, and enolase were also identified in chilling stress-related studies (Lee et al., 2009). Among them PPDk is responsible for the production of phosphoenolpyruvate (PEP), the primary acceptor of CO_2_(Moons et al., 1998); whereas aconitate hydratase, glycine dehydrogenase, and enolase are involved in the tricarboxylic acid cycle, photorespiration, and glycolysis (Hojoung et al., 2002; Cui et al., 2005), respectively. Similarly, higher abundance of adenylate kinase protein under chilling stress is an indication of enhanced ATP synthesis and energy metabolism. Additionally, peptidylprolyl isomerase Cyp2 and cysteine proteinase was preferentially accumulated in rice roots upon chilling stress (Pradet and Raymond, 1983; Hashimoto and Komatsu, 2007). Taken together, these results indicated that during cold stress plants require high energy production that comes from activation of metabolic pathways.

Quantitative analysis of PM proteome of rice roots grown under cold stress condition revealed that proteins related to membrane permeability and signal transduction through the membrane were enhanced in level (Hashimoto et al., 2009). Examples of this category include members of the annexin and hypersensitive-induced response (HIR) protein families. Annexins are Ca^2+^- dependent membrane binding proteins that play vital roles in membrane trafficking and organization, and are known to regulate ion channel activity and phospholipids metabolism (Gerke and Moss, 2002). Thus, elevated levels of annexins led to protection against the osmotic imbalance caused by cold stress. The HIR family of proteins belongs to a structurally-related superfamily, which includes prohibitins, stomatins, and other membrane proteins. These proteins control the ion channel activity and thereby regulate diverse cellular processes like cell division, osmotic homeostasis and cell death (Nadimpalli et al., 2000). Higher abundance of this family of proteins can also be explained as a response to osmotic stress induced by cold treatment. Other proteins that were found to be involved in the cold stress response include dehydrins, 25 KDa dehydrin-like protein, ERD14, and cold acclimation-specific protein 15 (CAS15) as identified in chicory roots under chilling stress (Degand et al., 2009). The first three proteins belong to the dehydrin family and are believed to form complexes with other macromolecules to protect the cells from freeze-induced desiccation (Kosová et al., 2007). CAS15 protein contains characteristic dehydrin K and S segments and thus contributes similarly to cold tolerance like dehydrins (Pennycooke et al., 2008).

On the other hand, cold stress causes oxidative damage to the cells by generating ROS or their precursors. To protect against this damaging effect, several anti-oxidants are produced in the root. For example, oxalyl-CoA decarboxylase, the second enzyme of oxalate catabolism pathway, was enriched in rice roots under chilling stress (Lee et al., 2009). This enzyme causes decarboxylation of activated oxalate molecule that generates ROS through fenton reaction. Unless decarboxylated, it causes generation of hydroxyl or carbonate radicals through its interaction with hydrogen peroxide (Urzúa et al. 1998). Gradual increase of glyoxalase I protein level throughout the cold stress period indicated detoxification of methylglyoxal produced during the stress condition (Espartero et al., 1995; Lee et al., 2009), thus providing another example of antioxidant generation. In addition, ROS scavengers induced commonly in all abiotic stresses, such as superoxide dismutase, catalase, and ascorbate peroxidase, were found in abundance in a study with chicory roots under chilling stress (Lee et al., 2009).

Moreover, heat shock proteins (HSPs) were found to be higher in abundance as a chilling stress response in roots of rice (Cui et al., 2005; Yan et al., 2006), chicory (Degand et al., 2009), maize (Kollipara et al., 2002), and poplar (Renaut et al., 2004), with HSP70 family being the most abundant. These proteins act as molecular chaperones and thus prevent aggregation of the denatured proteins as well as facilitate protein refolding (Lee et al., 2009). In addition, a putative calreticulin precursor with chaperone activity was also detected in a study with rice seedlings under chilling stress (Hashimoto and Komatsu, 2007). Defense-related proteins such as protein disulfide isomerase and disease resistance response protein were also detected in relatively high abundance in pea roots under chilling stress (Dumont et al., 2011) indicating that defense-related pathways are activated in the root when combating cold stress.

Several other proteome signatures provided insights on plants' preparation for recovery once the stress is being released. For example proteins involved in cellulose synthesis, such as UDP-glucose pyrophosphorylase were highly abundant in rice roots upon 48 h chilling stress (Amor et al., 1995; Hashimoto and Komatsu, 2007). This suggested new cell wall synthesis under chilling stress to confer enhanced protection of cells against low temperature.

## Conclusion

**Figure 2:**
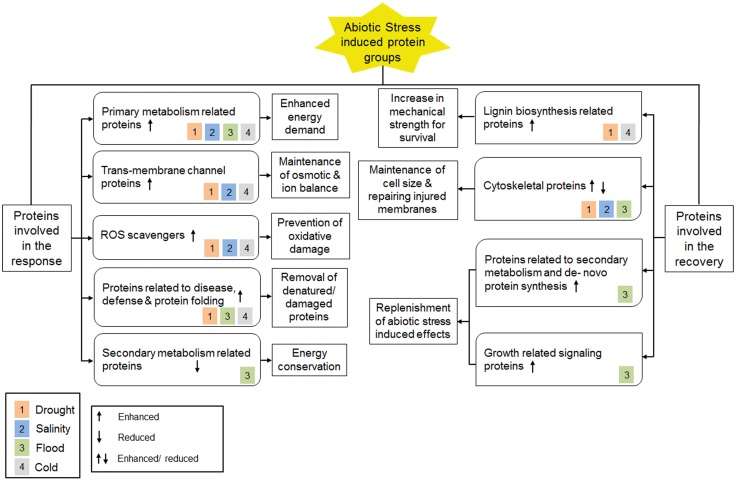
**A summary of proteome level changes detected under different abiotic stress conditions and current understanding of underlying molecular mechanisms**. Color blocks inside all protein classes represent different types of abiotic stress condition as shown in the legend.

Proteomic analyses on plant roots under various abiotic stress conditions revealed important information on proteins involved in the abiotic stress response. This leads to the identification of molecular and cellular mechanisms that are specific to certain abiotic stress or shared between two or more abiotic stress conditions (Figure 2). For instance, during drought, high salinity and cold stress conditions, trans-membrane water, and/or ion channel proteins were found to be higher in abundance, indicating changes in ion and/or osmotic balance. This phenomenon was however not observed in flooded conditions when the roots were submerged in water. In addition, higher abundance of ROS scavengers was detected in roots under drought, high salinity and cold stress and can be looked upon as a preventive measure against oxidative damage caused due to high ROS levels. By contrast, the abundance of ROS scavengers during flood condition was low, which can be explained as a fact that roots are maintained in a hypoxic state during flood. On the other hand, molecular chaperones involved in protein folding, disease-, and defense-related proteins, such as proteolytic enzymes and proteosomal factors, were found to have higher levels during drought, flood, and cold stress conditions, indicating refolding of denatured proteins and proteolytic elimination of damaged proteins. Moreover, all the abiotic stress conditions discussed in this review were shown to induce the protein levels involved in primary metabolism in the root, indicating an enhanced energy demand during the stress condition. However, secondary metabolism associated proteins were found to be low in abundance in roots during flood condition suggesting a mode of energy conservation.

At the recovery phase, increased lignin biosynthesis was found following cold and drought stress. This molecular mechanism results in enhanced mechanical strength of roots by hardening cell wall at the root tip. Changes in abundance for cytoskeleton associated proteins were also observed, that can be looked upon as compensation against reduced cell size as well as repairing injuries caused by drought, salinity, and flood stress. Moreover, the levels of proteins related to *de novo* protein synthesis, growth-related signaling, and secondary metabolism were enhanced during recovery from flood stress as a replenishment of the stress-induced effects. Together, these changes suggested a compensatory mechanism by which the stress-induced effects could significantly be recovered.

## Future perspectives

As mentioned in this review, majority of literature reported proteomic analyses of the root response at least after 24 h of exposure to cold, drought, high salinity, or flooding stress. Therefore, early proteomic changes associated with individual abiotic stress remain to be elucidated, which will allow the identification of distinct sets of effectors for stress signaling. The function of these early effectors may be masked by secondary processes at a later stage. Technically, most of these proteomic studies have utilized the gel based separation approaches (Table 1) that resulted in identification of mostly high abundant proteins. Use of advanced LC based separation techniques may significantly improve detectability of low abundant proteins such as transcription factors, kinases, and transport proteins. Additionally, post-translational changes such as phosphorylation, glycosylation, and oxidation, which are likely induced by the stressors, are still to be captured with the use of gel based proteomic approaches. Other than these aspects, changes in plant hormone-mediated metabolic programs, as well as alterations in protein-protein interactions and subcellular translocation of proteins, remain to be explored and correlated with different stressors. Future investigations on these areas are expected to improve our understanding of plant root responses to abiotic stress. These researches could be augmented with the use of comparatively new proteomic strategies such as hydrogen–deuterium exchange (H/D exchange), surface plasmon resonance (SPR)-MS together with integrated cell biology approaches such as immuno-precipitation and live imaging analysis. Since proteins are dynamic in nature and most published proteomic studies only focused on a single time point, carefully designed time-course experiments will thus be required in future to advance our knowledge on the time-dependent response or recovery mechanisms (Graves and Haystead, 2002).

### Conflict of interest statement

The authors declare that the research was conducted in the absence of any commercial or financial relationships that could be construed as a potential conflict of interest.
